# Functional brain networks associated with eating behaviors in obesity

**DOI:** 10.1038/srep23891

**Published:** 2016-03-31

**Authors:** Bo-yong Park, Jongbum Seo, Hyunjin Park

**Affiliations:** 1 Department of Electronic, Electrical and Computer Engineering, Sungkyunkwan University, Korea; 2 Department of Biomedical Engineering, Yonsei University, Korea; 3 School of Electronic Electrical Engineering, Sungkyunkwan University, Korea; 4 Center for Neuroscience Imaging Research (CNIR), Institute for Basic Science, Korea

## Abstract

Obesity causes critical health problems including diabetes and hypertension that affect billions of people worldwide. Obesity and eating behaviors are believed to be closely linked but their relationship through brain networks has not been fully explored. We identified functional brain networks associated with obesity and examined how the networks were related to eating behaviors. Resting state functional magnetic resonance imaging (MRI) scans were obtained for 82 participants. Data were from an equal number of people of healthy weight (HW) and non-healthy weight (non-HW). Connectivity matrices were computed with spatial maps derived using a group independent component analysis approach. Brain networks and associated connectivity parameters with significant group-wise differences were identified and correlated with scores on a three-factor eating questionnaire (TFEQ) describing restraint, disinhibition, and hunger eating behaviors. Frontoparietal and cerebellum networks showed group-wise differences between HW and non-HW groups. Frontoparietal network showed a high correlation with TFEQ disinhibition scores. Both frontoparietal and cerebellum networks showed a high correlation with body mass index (BMI) scores. Brain networks with significant group-wise differences between HW and non-HW groups were identified. Parts of the identified networks showed a high correlation with eating behavior scores.

More than two billion adults worldwide are overweight and have obesity-related health problems such as diabetes, hypertension, and stroke^1^,^2^. Complex genetic, environmental and behavior factors affect obesity^3^. Poor eating behavior is one of the important factors that cause obesity and thus many studies have explored the relationship between eating behavior and obesity^3^,^4^. Poor eating behavior is strongly linked to higher body mass index (BMI), which is a critical factor that promote weight gain^3^,^4^. Conventional studies linked obesity with eating behavior through modulation in hormones such as letpin and ghrelin^5^,^6^. Leptin and ghrelin are known to be related to BMI and body fat^5^,^6^. Abnormal secretion of leptin and ghrelin changes the brain reward systems and promote overeating^5^,^6^. Impulsivity and inhibitory control were found to be related to eating behavior^7^. People with high impulsivity and low inhibitory control showed tendency to overeat and were more likely to be obese^7^,^8^,^9^,^10^. Elevated impulsivity and reduced inhibitory control were positively correlated with BMI and disinhibition in eating^7^,^10^,^11^. Eating behavior is also believed to be linked with brain networks besides factors such as hormone modulation, impulsivity and inhibitory control^1^. Reward networks including the orbitofrontal cortex and insula are the most affected by eating behaviors^12^,^13^,^14^,^15^. Dysfunction in the prefrontal and parietal cortex changes the reward network and promotes weight gain^1^. Cognitive neuronal systems including the dorsolateral prefrontal cortex, insula, and inferior parietal cortex regulate appetite responses^16^,^17^,^18^,^19^. Alterations in the cognition system disrupt the balance between reward and the cognition system and might lead to abnormal eating behaviors^1^,^15^. Errant eating behaviors strongly affect the brain reward and cognition systems and therefore could even be considered as a disease^1^,^12^,^15^,^16^,^17^,^18^,^19^. The relationship between eating behavior and brain networks has not been fully explored. The objective of our study was to find the relationship between eating behavior and brain networks in obesity. Eating behavior can be measured by the three-factor eating questionnaire (TFEQ)^20^. The three TFEQ factors cover restraint (TFEQ-R), disinhibition (TFEQ-D), and hunger (TFEQ-H)^20^.

Neuroimaging is a widely adopted, noninvasive tool for assessing brain networks. Studies have adopted various neuroimaging techniques to assess brain networks in people with obesity using magnetic resonance imaging (MRI), single-photon emission computed tomography, and positron emission tomography^15^,^21^,^22^. MRI is especially useful as it obtains both structural and functional information. Resting-state functional MRI (rs-fMRI) measures local brain activity using the blood oxygen level-dependent effect, which we adopted for this study.

Several brain regions form networks that interact and share functions^23^. Several studies adopted network analysis to identify interactions between brain networks in people with obesity^13^,^15^,^24^,^25^. However, links between eating behavior and obesity in terms of brain networks has not been fully explored. The connection between eating behaviors and brain functions might be better explained using brain networks. Connectivity analysis observes the entire brain as a complex interconnected network, focusing on how activities in one region correlate with activities in another region^23^,^26^. Connectivity information is measured with graph structure with nodes and edges. Nodes were computed with spatial maps derived from group independent component analysis (ICA), a data-driven method with better sensitivity to quantify functional connectivity than conventional region-based methods^27^,^28^. We adopted weighted edges instead of threshold edges to fully incorporate the full spectrum of edge values to better quantify brain networks^29^,^30^.

We aimed to (1) find spatial maps derived from a group ICA approach and weighted edge values, (2) identify group-wise brain network differences in degree centrality, a graph theoretical measure, between HW and non-HW groups, and (3) quantify the relationship between brain networks and eating behavior using identified networks, associated degree values and TFEQ scores. We hypothesized that cognition network might show connectivity differences between HW and non-HW group, and might correlate significantly with eating behavior.

## Results

### Spatial maps from group ICA

**Figure 1:**
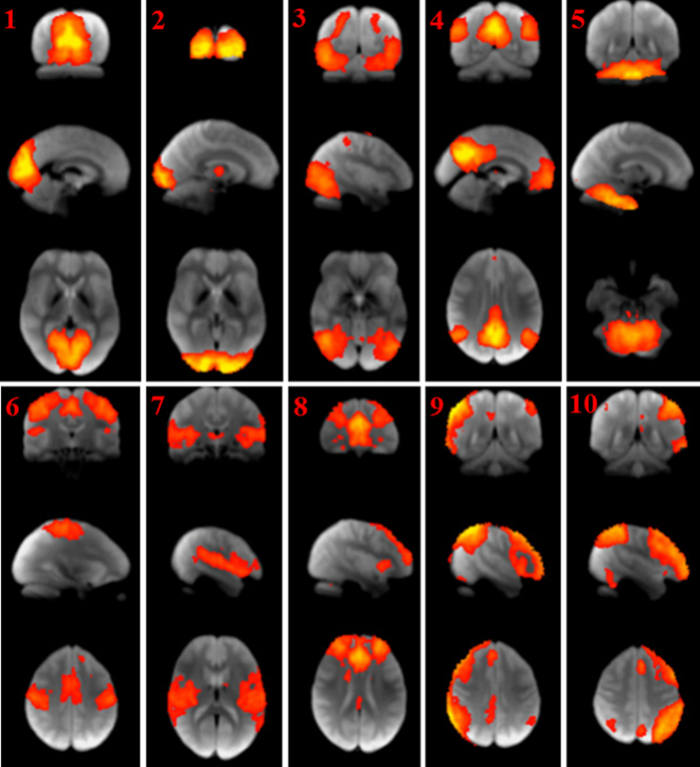
Ten sample reference RSNs. RSNs 1, 2 and 3 correspond to a visual network. RSN 4 corresponds to a default mode network. RSN 5 corresponds to the cerebellum. RSN 6 corresponds to a sensorimotor network. RSN 7 corresponds to an auditory network. RSN 8 corresponds to an executive control network. RSN 9 and 10 correspond to a frontoparietal network. Further details are available^31^.

Group ICA approach automatically generated spatial maps, called independent component (ICs) using all subjects’ time series data. The number of ICs was not pre-determined but was computed by a data-driven approach. Networks of obtained ICs were defined by comparing ICs with known fMRI resting state networks (RSNs) (see the Methods section). The group ICA approach generated 48 components that were spatial maps or ICs (Supplementary Fig. S1). Cross-correlation between generated ICs and reference RSNs (Fig. 1) showed 14 ICs with significant correlation to reference RSNs (Supplementary Table S1 and Supplementary Table S2)^31^. Reference RSNs 1, 2, and 3 (generated IC 2, 18, and 4) covered a visual network of the bilateral superior-, middle-, and inferior occipital gyrus; lingual gyrus; and cuneus. Reference RSN 4 (generated IC 5 and 24) covered part of a default mode network of the bilateral precuneus, middle- and posterior-cingulate cortex; and angular gyrus. Reference RSN 5 (generated IC 38) covered the cerebellum; and vermis. Reference RSN 6 (generated IC 19) covered the sensorimotor network of bilateral precentral gyrus; and postcentral gyrus. Reference RSN 7 (generated IC 10 and 22) covered the auditory network of bilateral superior temporal gyrus; insula; Heschl’s gyrus; and rolandic operculum. Reference RSN 8 (generated IC 3, 7, and 26) covered executive control network including bilateral medial-, superior-, and inferior-frontal gyrus; anterior-, middle-, and posterior-cingulate cortex; and thalamus. Reference RSN 9 and 10 (generated IC 25 and 27) covered the frontoparietal network of the bilateral middle orbitofrontal gyrus; inferior frontal gyrus; superior- and inferior-parietal lobule; and supramarginal gyrus.

### Differences in connectivity

Weighted degree values were computed for each IC to assess group-wise connectivity differences between HW and non-HW groups (see the Methods section). Two ICs including the frontoparietal and cerebellum networks showed significant differences (*p* < 0.05, corrected) (Supplementary Table S3). The effect sizes of the identified ICs were calculated using Cohen’s d formula^32^. The effect sizes of frontoparietal network and cerebellum network were 0.4861 and 0.5426 respectively (Supplementary Table S3). The effect size values might be interpreted as having a moderate statistical significance^32^. IC 25 covered the frontoparietal network and IC 38 covered the cerebellum network.

### Correlation between clinical variables and degree value

**Figure 2:**
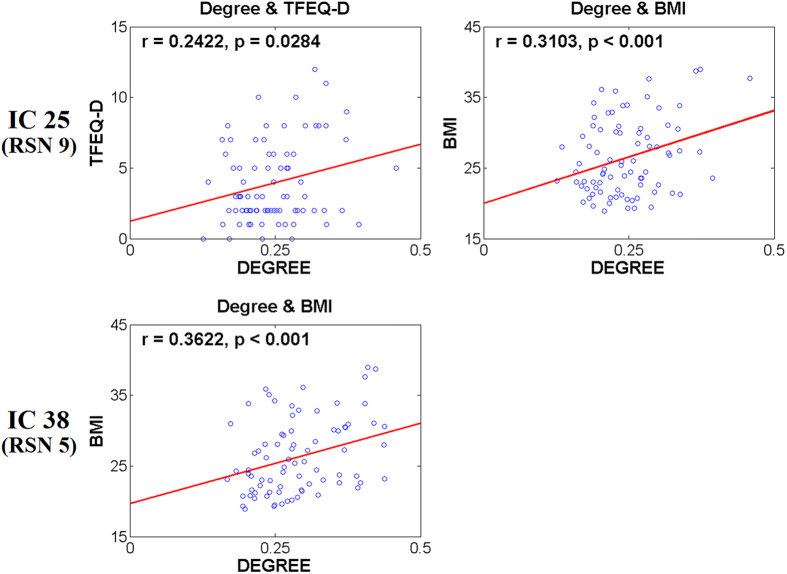
Correlation between degree values of identified ICs and TFEQ and BMI scores. RSN index numbers along with IC index numbers are displayed. Only the correlation with TFEQ-D scores is reported as other correlations did not show significant results.

Correlation between degree values of two identified ICs and TFEQ and BMI scores were investigated (Supplementary Table S4). Frontoparietal network (IC 25) showed significant correlation with TFEQ-D scores (*r* = 0.2422, *p* = 0.0284). Both the frontoparietal and cerebellum networks (IC 25 and 38) significantly correlated with BMI scores (*r* = 0.3103, *p* < 0.001; *r* = 0.3622, *p* < 0.001, respectively). Significant correlation results are shown in Fig. 2.

## Discussion

The main objective of this study was to find brain networks related to eating behaviors and obesity based on neuroimaging analysis. Our study quantified brain network differences of HW and non-HW groups using ICs generated by group ICA approach and associated weighted degree values. Two ICs involved in frontoparietal and cerebellum networks showed significant connectivity differences between HW and non-HW groups. In particular, frontoparietal network showed significant (*p* < 0.05) correlation with TFEQ-D scores (*r* = 0.2422; *p* = 0.0284). Both the frontoparietal and cerebellum networks showed high correlation with BMI (*r* = 0.3103, *p* < 0.001; *r* = 0.3622, *p* < 0.001, respectively). The correlation between degree values of identified ICs and BMI ensured that identified ICs were consistent with general obesity characteristics.

Many studies focused on finding metabolic mechanism to link obesity and eating disorder^5^,^6^. A few studies attempted to relate eating disorder with neuroimaging^1^,^13^. Val-Laillet, D. *et al*. explored the relationship between eating disorders and obesity by observing the altered activations in reward regions when external stimuli were applied^1^. Coveleskie, K. *et al*. compared lean subjects and people with obesity using seed based functional connectivity^13^. Existing studies did not consider ICA based functional connectivity analysis^1^,^13^. The relationship between eating behavior and brain networks has not been fully explored. Our study attempted to find brain networks related to eating behavior in obesity. We believe it might provide cues for future obesity and eating behavior related research. In our study, the frontoparietal and cerebellum networks showed significant connectivity differences between HW and non-HW groups. Frontoparietal network regulate reward and cognitive functions^13^,^15^,^24^,^25^,^31^. Previous studies showed the reward and cognitive systems are highly associated with appetite and eating behavior^1^,^18^. Low activity in the frontoparietal network confuses the processes of satiety and leads to greater sensitivity to food intake^1^. Enhanced activation in dorsolateral prefrontal cortex confuses the reward and cognitive system and might cause overeating^1^. Cerebellum was considered as a motor center, still a few studies demonstrated that it is related to eating behaviors and weight change^33^,^34^. Cerebellum showed different activations between obese and lean subjects^33^.

There are several network centrality measures such as degree-, betweenness-, eigenvector-, and closeness-centrality^30^. All centrality measures reflect the importance of a given node^30^. There are slight differences among centrality measures and different studies have adopted different centrality measures^35^,^36^. There is no single best centrality measure that is guaranteed to perform well for various research questions^37^,^38^. The computation efficiency of degree centrality is higher than other centrality measures because it requires only the edge weights^30^. In addition, degree centrality has been known to be sensitive to local changes in brain connectivity^26^,^30^. We adopted degree centrality over other centrality measures as it is a simple and sensitive local parameter to describe the complex brain network. Following studies adopted different centrality measures to quantify brain connectivity to suit their needs^35^,^36^. Seo *et al*. compared brain networks between normal subjects, mild cognitive impairment (MCI) patients, and Alzheimer’s disease (AD) patients using betweenness centrality^36^. Xia *et al*. defined network hubs in normal subjects and attention deficit hyperactivity disorder (ADHD) patients using degree- and betweenness-centrality^35^.

ICA approach generates spatial maps, a set of voxels sharing similar brain activity patterns^39^,^40^. Roughly put, ICA maps could be thought as functional parcellation of the brain. Many neuroimaging studies used anatomically pre-defined regions such as automated anatomical labeling (AAL) to compare two groups^35^,^36^,^41^,^42^. These studies would quantify group differences on anatomically defined regions. Studies adopting ICA approach would quantify group differences on functionally defined regions and thus are better suited to functional imaging studies^39^,^40^. In addition, ICA maps could be easily related to known brain networks and thus easier to interpret^31^. Group-wise difference in ICA maps relates to difference between sets of functionally defined voxels not difference between sets of anatomically defined voxels^39^,^40^.

The degree values of identified ICs correlated with clinical variables of eating behavior and obesity characteristics (TFEQ and BMI scores). ICs with significant correlation to TFEQ scores could be functional correlates associated with eating behaviors. Frontoparietal network showed significant (*p* < 0.05) correlation with TFEQ-D scores. TFEQ-D score reflect a tendency to overeat and high TFEQ-D scores are related to obesity^43^,^44^. Our results might indicate that cognition network associated with obesity are also highly correlated with eating disinhibition, as shown in previous studies^1^,^12^,^17^. In addition, both the frontoparietal and cerebellum networks showed the high correlation with BMI. Frontoparietal and cerebellum networks are related to obesity and moreover frontoparietal network has strong association with eating behavior^1^,^18^,^33^. Our results were consistent with previous studies^1^,^12^,^17^,^18^,^33^.

Our study has several limitations. First, we adopted degree centrality as a network measure to quantify differences in connectivity. Other network features including betweenness-, eigenvector-, and closeness-centrality might be more suitable for obesity-related brain networks^26^,^30^. Second, our study focused only on rs-fMRI. Multimodal analysis such as combining rs-fMRI and diffusion weighted imaging might provide complementary information to better quantify the complex brain network. Finally, a longitudinal study that follows weight gain or loss is needed to assess the stability of our findings.

Our study showed that brain dysfunction in cognition network was related to errant eating behavior and obesity. Brain networks are important for obesity as they are regulated by hormones including leptin and ghrelin that control appetite^5^,^6^. We found shared brain networks correlated with obesity and eating behavior. The identified shared brain networks might be important image biomarkers for obesity-related research. We propose that future research on obesity and abnormal eating behaviors should consider brain network alterations.

## Methods

### Subjects and imaging data

Institutional Review Board (IRB) of Sungkyunkwan University approved our retrospective study. Our study was performed in full accordance with the local IRB guidelines. Informed consent was obtained from all subjects. We collected rs-fMRI and T1-weighted structure data from the Nathan Kline Institute/Rockland Sample database^45^. Rs-fMRI data were acquired on a Siemens Magnetom Trio Tim scanner with the following imaging parameters: number of slices = 38; slice thickness = 3 mm; pixel resolution = 3 mm isotropic; repetition time = 2500 ms; echo time = 30 ms; and field of view = 216 mm. T1-weighted structure images were acquired with following imaging parameters: number of slices = 192; pixel resolution = 1 mm isotropic; repetition time = 2500 ms; echo time = 3.5 ms; and field of view = 256 mm. The phase encoding of rs-fMRI and T1-weighted structural images was from anterior to posterior direction. Eight underweight participants with BMI less than 18.5 and one participant with unknown BMI were excluded from the total of 166 participants. 58 participants with psychiatric conditions related to attention, withdrawn, somatic, thoughtful, anxious/depression, rule breaking, aggressive, and intrusive problems were excluded based on adult self-report (ASR) scores^46^. The remaining participants were classified into 41 HW and 58 non-HW subjects. HW and non-HW groups were classified using body mass index (BMI), a measure based on height and weight. 17 non-HW subjects were excluded to match sex ratio and age between HW and non-HW groups. Finally, 41 HW and 41 non-HW subjects were considered for the study. Sex ratios and age between groups did not show significant differences (*p* > 0.05). BMI and TFEQ scores showed significant differences (*p* < 0.05) between groups (Table 1). Detailed participant information is given in Table 1.

### Image preprocessing

All rs-fMRI data were preprocessed using FSL software with standardized preprocessing procedures^47^. Skull was removed using BET. Magnetic field bias was corrected and brain tissues were classified into white or gray matter and cerebrospinal fluid with FAST. Head motion was corrected using MCFLIRT. Slice timing correction was performed to align slices with different timing using SLICETIMER. Spatial smoothing was applied with full width at half maximum value of 6 mm. Intensity normalization of time series 4D data was applied with a value of 10,000. A high-pass filter with cutoff 100 second was applied. We did not apply band-pass filtering and only kept high frequency signals based on recent findings^48^,^49^. Functional EPI images were registered onto the T1-weighted structural image and T1-weighted structural image was subsequently registered onto the Montreal Neurological Institute standard space. The 4D dataset was resampled to 3 mm isotropic resolution.

### Group ICA

All subjects’ time series data were temporally concatenated and fed into FSL MELODIC software^39^. An ICA approach was applied to generate spatial maps automatically, called independent components (ICs)^40^. The process of determining number of IC was driven by data. Each spatial map was a collection of voxels sharing similar patterns of brain activity. Obtained ICs were used as regressors to estimate participant-specific time series^50^. The generated ICs contain functionally interpretable networks as well as uninteresting signals. Obtained ICs were compared with known fMRI resting state networks (RSNs)^31^. Establishing such connection to reference RSNs allowed standardized interpretation of results^31^. Cross correlations between the obtained ICs and reference RSNs were calculated with threshold of 0.35, which was a stricter criterion than that of a previous study, 0.25 31. The process ensured that only functionally interpretable ICs were kept after the cross correlation procedure.

### Network construction

Connectivity analysis requires the regions of interest (ROIs) to investigate correlation between different regions. We considered functionally interpretable ICs generated from ICA approach. Each IC was represented as a node in a graph. Each edge was defined as the correlation of the time series between two different nodes. We adopted the weighted and undirected network model. Edge values were entered into the matrix as elements and the matrix was referred to the correlation matrix. Soft thresholding was applied to weights to avoid binarizing the correlation matrix using the following equation; 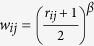, where *r*_*ij*_ means the edge value between the node *i* and *j*^51^,^52^. The *β* value was set to twelve to ensure scale-free topology^51^. The soft thresholded correlation matrix was *z*-transformed using Fisher’s *r*-to-*z* transform. Network construction was performed using MATLAB (Mathworks Inc., USA).

### Connectivity analysis

There are several network centrality measures such as degree-, betweenness-, eigenvector-, and closeness-centrality^30^. Degree centrality is defined as the sum of all edge weights connected to a given node^30^. Betweenness centrality is defined as the number of shortest paths between any two nodes that run through that node^30^. Eigenvector centrality of node *i* is defined as the *i*^*th*^ element in the eigenvector corresponding to the largest eigenvalue of the correlation matrix^52^,^53^. It considers neighborhood nodes as well the given node itself^52^,^53^. Thus it is a locally weighted centrality measure. Edges with high node centrality contribute more to the network in using eigenvector centrality^52^,^53^. Closeness centrality is defined as the inverse of the average shortest path length from one node to all other nodes^30^. Degree centrality is one of the sensitive network measures among measures such as betweenness-, eigenvector-, and closeness centrality^30^. We adopted degree centrality because it is a simple and sensitive local parameter to describe the brain network^30^,^37^. Degree centrality was computed as weighted degree in the weighted network model. The weighted degree value is the sum of all edge weights connected to a given node^26^. A node (i.e., chosen IC) with high degree centrality refers to an important node where the strength of information flow is high^30^. Degree values for each node (i.e., ICs) were compared between HW and non-HW groups and ICs showing group-wise differences were identified. Connectivity analysis was performed using MATLAB (Mathworks Inc., USA).

### Correlation with clinical variables

Degree values of identified ICs were correlated with clinical variables including three TFEQ and BMI scores. The correlation was performed to determine if the identified ICs were related to eating behavior and obesity characteristics (TFEQ and BMI). A general linear regression model was applied: clinical score = *α* + *β* · degree centrality, where *α* was a constant and *β* was the estimated coefficient.

### Statistical analysis

Differences between HW and non-HW groups were assessed performing permutation tests 10,000 times randomly assigning participants to HW and non-HW groups to avoid multiple comparison issue^27^. The null distribution was constructed from the permutation tests. Statistically significant ICs were identified if the degree values of the ICs did not belong to the 95% of the null distribution (p < 0.05, corrected). The effect sizes were calculated with degree values using Cohen’s d formula^32^. Cohen’s d was calculated by dividing the difference of mean values between two groups by a pooled standard deviation as follows: 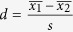, where *s* is the pooled standard deviation of two groups and 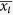 is the mean degree value of group *i*^32^. Cohen’s d can be either positive or negative according to the mean values of each group. The magnitude of Cohen’s d reflects the significance of the group differences not necessarily the sign of d. The magnitude of the Cohen’s d larger than 0.8 is considered as a high statistical significance, around 0.5 is considered as a moderate statistical significance, and lower than 0.2 is considered as a low statistical significance according to established previous studies^32^. Higher d values lead to lower p-values. The identified ICs and the associated degree values were correlated with clinical scores. The significance of the linear regression between clinical scores and degree values were quantified with *r*- and *p*-values. All statistical analyses were performed using MATLAB (Mathworks Inc., USA).

## Additional Information

**How to cite this article**: Park, B.-y. *et al*. Functional brain networks associated with eating behaviors in obesity. *Sci. Rep*. **6**, 23891; doi: 10.1038/srep23891 (2016).

## Supplementary Material

Supplementary Information

## Figures and Tables

**Table 1:** Demographic data of HW and non-HW groups (means and standard deviations [SD]).

Information	HW (n = 41)	Non-HW (n = 41)	*p*-value
Gender (M:F)	25:16	25:16	1
Age	29.83 (9.95)	33.24 (10.09)	0.1269
BMI	22.03 (1.67)	30.88 (3.82)	<0.001
TFEQ-R	6.41 (4.60)	8.51 (4.58)	0.0418
TFEQ-D	3.20 (2.40)	4.61 (3.08)	0.0229
TFEQ-H	2.93 (2.09)	5.61 (3.44)	<0.001

HW, healthy weight; M, male; F, female; BMI, body mass index; TFEQ-R, three-factor eating questionnaire restraint; TFEQ-D, three-factor eating questionnaire disinhibition; TFEQ-H, three-factor eating questionnaire hunger.
