# Supplementary material for: Functional brain networks associated with eating behaviors in obesity
Source: Sci Rep. 2016 Mar 31;6:23891. doi: 10.1038/srep23891 (PMC4814917; doi:10.1038/srep23891)
Supplement: Supplementary Information [file srep23891-s1.pdf]

Supplementary Information

Title: Functional brain networks associated with eating behaviors in obesity

Running title: Functional brain networks of eating behaviors

Authors: Bo-yong Park[1], Jongbum Seo[2], and Hyunjin Park[3,4]*

1) Department of Electronic, Electrical and Computer Engineering, Sungkyunkwan University, Korea

2) Department of Biomedical Engineering, Yonsei University, Korea

3) School of Electronic Electrical Engineering, Sungkyunkwan University, Korea

4) Center for Neuroscience Imaging Research (CNIR), Institute for Basic Science, Korea

*Corresponding Author:

Hyunjin Park, PhD

School of Electronic Electrical Engineering

Sungkyunkwan University, Korea

Phone: +82-31-299-4956

Fax: +82-31-290-5819

Email: hyunjinp@skku.edu

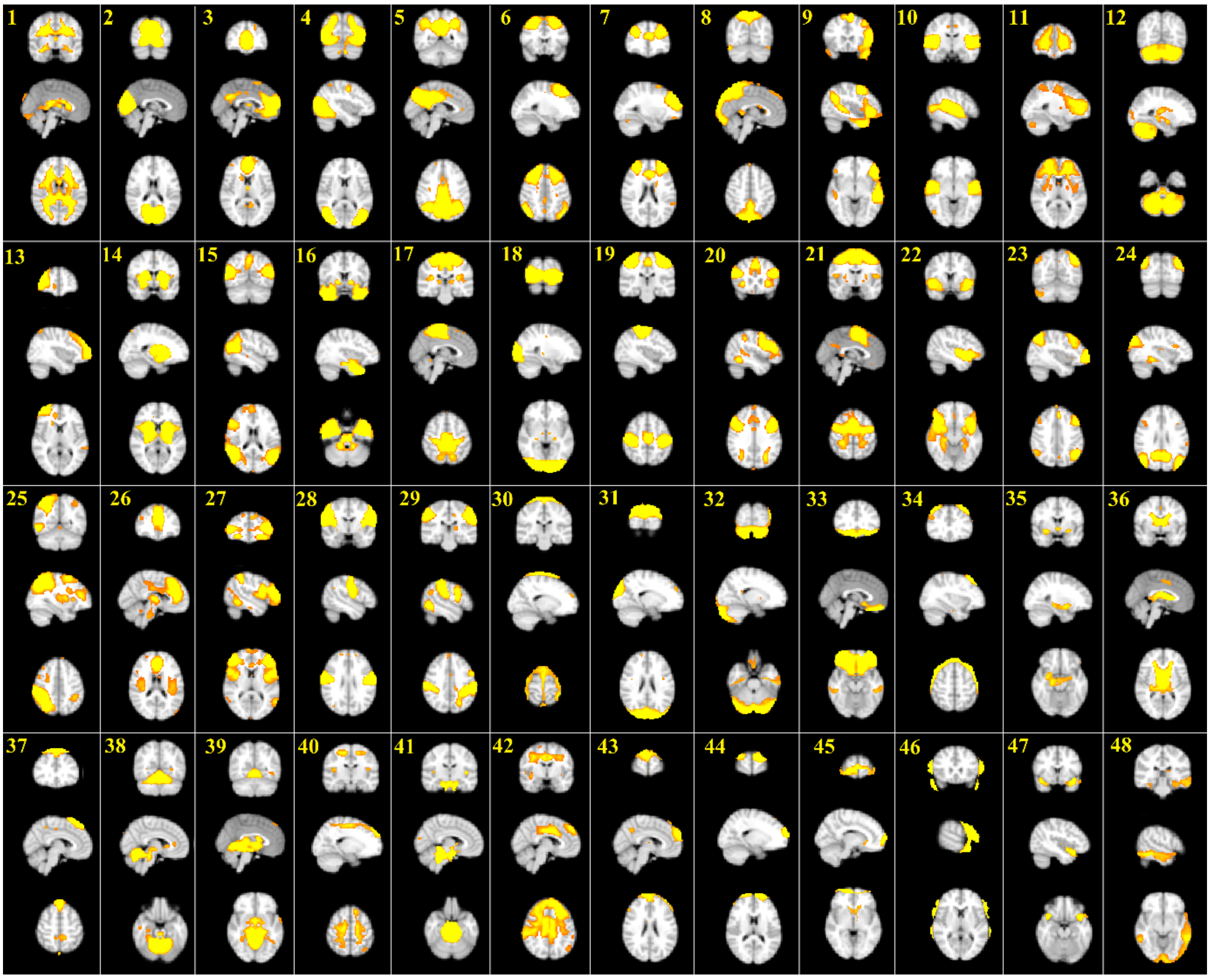

Supplementary Figure S1. The 48 automatically generated ICs by group ICA. All ICs were threshold at z statistic images with $p > 0.5$.

Supplementary Table S1. Correlation between 14 significant, generated ICs and reference RSNs. Some reference RSNs showed significant correlation with several generated ICs as seen in reference RSNs 4, 7 and 8.

Reference ICs	Generated ICs	*r*-value
1	2	0.84
2	18	0.77
3	4	0.73
4	5, 24	0.59, 0.49
5	38	0.50
6	19	0.41
7	10, 22	0.59, 0.43
8	3, 7, 26	0.42, 0.39, 0.37
9	25	0.42
10	27	0.36

ICs, independent components

Supplementary Table S2. Fourteen generated ICs significantly correlated with reference RSNs. Correlation values had mean $r = 0.52$ and range 0.36–0.84. Yellow numbers, upper left: identified index number of generated ICs; red numbers, lower left: identified index number of reference RSNs.

Network	Region	ICs
Visual	Superior occipital gyrus<br>Middle occipital gyrus<br>Inferior occipital gyrus<br>Lingual gyrus<br>Cuneus	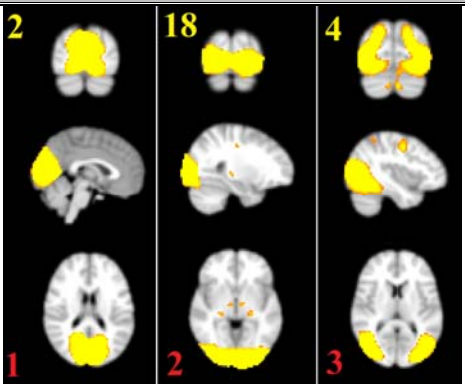
Default mode	Precuneus<br>Middle cingulate cortex<br>Posterior cingulate cortex<br>Angular gyrus	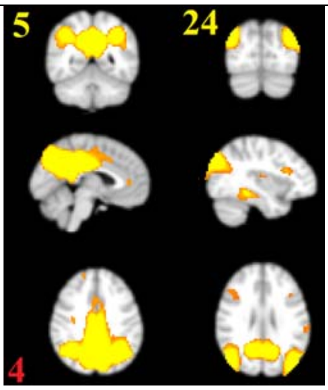
Cerebellum	Cerebellum<br>Vermis	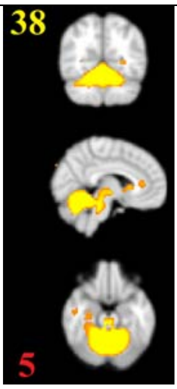

Network	Region	ICs
Sensorimotor	Precentral gyrus<br>Postcentral gyrus	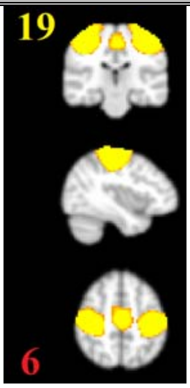
Auditory	Superior temporal gyrus<br>Insula<br>Heschl’s gyrus<br>Rolandic operculum	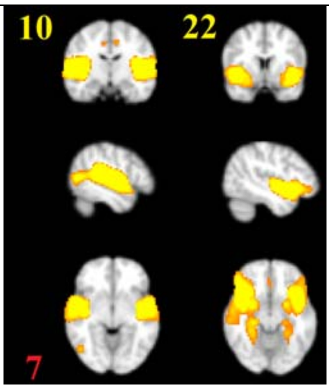
Executive control	Medial frontal gyrus<br>Superior frontal gyrus<br>Inferior frontal gyrus<br>Anterior cingulate cortex<br>Middle cingulate cortex<br>Posterior cingulate cortex<br>Thalamus	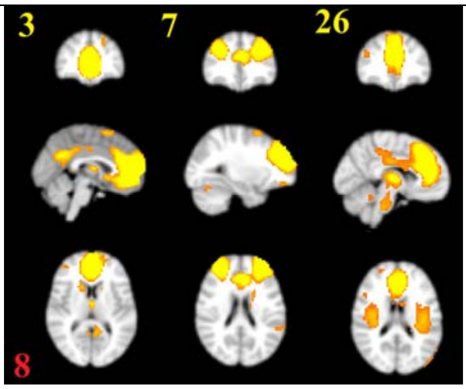
Frontoparietal	Middle orbitofrontal gyrus<br>Inferior frontal gyrus<br>Superior parietal lobule<br>Inferior parietal lobule<br>Supramarginal gyrus	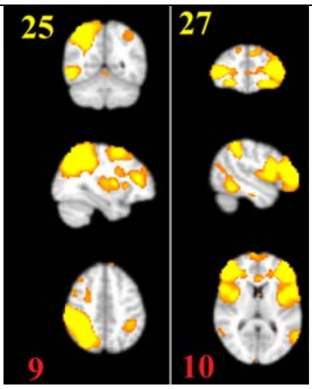

Supplementary Table S3. Identified ICs with significant group-wise connectivity differences between HW and non-HW groups based on weighted degree measure.

IC number	Network	*p*-value, corrected	Effect size
25	Frontoparietal	0.0318	0.4861
38	Cerebellum	0.0018	0.5426

ICs, independent components

Supplementary Table S4. Correlation between degree values of identified ICs and clinical variables. Significant results are reported in italicized bold font.

IC	TFEQ-R		TFEQ-D		TFEQ-H		BMI	
	r	***p***	***r***	***p***	***r***	***p***	***r***	***p***
25	0.1015	0.3642	***0.2422***	***0.0284***	0.1920	0.0841	***0.3103***	***0.0046***
38	-0.0884	0.4298	-0.0060	0.9572	0.0929	0.4064	***0.3622***	***0.0008***

IC, independent component; TFEQ-R, three-factor eating questionnaire restraint; TFEQ-D, three-factor eating questionnaire disinhibition; TFEQ-H, three-factor eating questionnaire hunger; BMI, body mass index
